# Impaired dendritic growth and positioning of cortical pyramidal neurons by activation of aryl hydrocarbon receptor signaling in the developing mouse

**DOI:** 10.1371/journal.pone.0183497

**Published:** 2017-08-18

**Authors:** Eiki Kimura, Ken-ichiro Kubo, Toshihiro Endo, Wenting Ling, Kazunori Nakajima, Masaki Kakeyama, Chiharu Tohyama

**Affiliations:** 1 Laboratory of Environmental Health Sciences, Center for Disease Biology and Integrative Medicine, Graduate School of Medicine, The University of Tokyo, Tokyo, Japan; 2 Center for Health and Environmental Risk Research, National Institute for Environmental Studies, Tsukuba, Japan; 3 Research Fellow of Japan Society for the Promotion of Science, Tokyo, Japan; 4 Department of Anatomy, Keio University School of Medicine, Tokyo, Japan; 5 Department of Neurochemistry, Graduate School of Medicine, The University of Tokyo, Tokyo, Japan; 6 Laboratory for Systems Neuroscience and Preventive Medicine, Faculty of Human Sciences, Waseda University, Tokorozawa, Japan; 7 Department of Anatomy and Embryology, Faculty of Medicine, University of Tsukuba, Tsukuba, Japan; Osaka University, JAPAN

## Abstract

The basic helix-loop-helix (bHLH) transcription factors exert multiple functions in mammalian cerebral cortex development. The aryl hydrocarbon receptor (AhR), a member of the bHLH-Per-Arnt-Sim subfamily, is a ligand-activated transcription factor reported to regulate nervous system development in both invertebrates and vertebrates, but the functions that AhR signaling pathway may have for mammalian cerebral cortex development remains elusive. Although the endogenous ligand involved in brain developmental process has not been identified, the environmental pollutant dioxin potently binds AhR and induces abnormalities in higher brain function of laboratory animals. Thus, we studied how activation of AhR signaling influences cortical development in mice. To this end, we produced mice expressing either constitutively active-AhR (CA-AhR), which has the capacity for ligand-independent activation of downstream genes, or AhR, which requires its ligands for activation. In brief, CA-AhR-expressing plasmid and AhR-expressing plasmid were each transfected into neural stems cells in the developing cerebrum by *in utero* electroporation on embryonic day 14.5. On postnatal day 14, mice transfected *in utero* with CA-AhR, but not those transfected with AhR, exhibited drastically reduced dendritic arborization of layer II/III pyramidal neurons and impaired neuronal positioning in the developing somatosensory cortex. The effects of CA-AhR were observed for dendrite development but not for the commissural fiber projection, suggesting a preferential influence on dendrites. The present results indicate that over-activation of AhR perturbs neuronal migration and morphological development in mammalian cortex, supporting previous observations of impaired dendritic structure, cortical dysgenesis, and behavioral abnormalities following perinatal dioxin exposure.

## Introduction

The mammalian cerebral cortex consists of six layers, each of which harbors subsets of neurons distinguished by morphology and synaptic organization: each layer has specific pyramidal neurons that are endowed with characteristic dendritic arborization and axonal projection patterns [1, 2]. Development of the cortex consists of a series of processes that are characterized by sequential progenitor proliferation, neuronal migration, and dendritic and axonal growth, leading to the establishment of functional cortical circuits required for higher brain function. These developmental processes are regulated by molecular signaling pathways that consist of ligand-receptor interactions and transcription factor activation [3, 4].

The basic helix-loop-helix (bHLH) transcription factors possess multiple functions in mammalian brain development. They can be divided into three main subfamilies: (1) the bHLH domain only (bHLH only), (2) bHLH domain contiguous with a leucine zipper (bHLH-Zip), and (3) bHLH domain contiguous with a Per-Arnt-Sim (PAS) domain (bHLH-PAS) [5, 6]. Members of these subfamilies exhibit region- and cell type-specific effects on progenitor proliferation, neuronal differentiation, and corticogenesis. The bHLH only subfamily contains members, such as neurogenin (Ngn), neurogenic differentiation (Neurod), and hairy and enhancer of split (Hes). Phosphorylated Ngn2 regulates the migration and dendritic morphology of mammalian cortical neurons [7, 8]. *Neurod1*-null mice show histological degeneration of the hippocampus and cerebellum [9]. Hes1 and Hes5 are highly expressed in neural stem cells, and inhibit differentiation into mature neurons [10, 11]. c-Myc and N-Myc, which belong to the bHLH-Zip subfamily, control cellular proliferation in the cerebrum and cerebellum [12, 13]. Sim1 and Sim2, members of the bHLH-PAS subfamily, have been reported to regulate neuronal subtype specification in the hypothalamus [14, 15]. Hence, clarification of the mechanism of mammalian cortical development requires a complete understanding of the functions of the bHLH family genes.

The aryl hydrocarbon receptor (AhR), which belongs to the bHLH-PAS subfamily, is expressed in the mammalian brain, including the cerebral cortex in the developing and adult stages [16–18], and acts as a receptor for ligands to induce downstream signaling. The liganded AhR is activated by cofactors and translocated from the cytoplasm to the nucleus, where it enhances the transcription of target genes such as *Cyp1a1*, *Cyp1b1*, and *Ahr repressor* (*Ahrr*) [19, 20]. Although the endogenous AhR ligand that contributes to neurodevelopment has not been identified, loss- or gain-of-function experiments have demonstrated important roles of AhR in nervous system development. In *Caenorhabditis elegans*, *ahr-1*, a homolog of the mammalian AhR, regulates neuronal migration, axonal branching, and neuronal subtype specification [21, 22]. *Spineless*, the *Drosophila* homolog of the mammalian AhR, controls the complexity of dendritic arborization in sensory neuron subtypes [23]. In the mouse cerebellum, AhR has been shown to regulate granule cell neurogenesis [24]. Thus, it is plausible that AhR-mediated signaling regulates neuronal differentiation and maturation in the mammalian cerebral cortex.

Dioxin, a persistent and highly toxic environmental contaminant, is an extremely potent exogenous AhR ligand, and activation of AhR signaling by dioxin has been shown to induce a variety of toxic effects, such as cancer, reproductive toxicity, and immunotoxicity [25]. Studies on AhR-null mice have demonstrated that the AhR is necessary for these toxic effects of dioxin [26–28]. In addition, adult rodent offspring born to dams exposed to dioxin during gestation exhibit cognitive and behavioral abnormalities, such as spatial memory [29], fear conditioning [30, 31], operant responses [32, 33], paired-association learning [34], and impaired flexibility and social behavior [35]. Additionally, *in utero* and lactational dioxin exposure induces the expression of AhR target genes [36] and disrupts dendritic morphology in the developing mouse brain [37]. These exposure experiments suggest that AhR signaling is indeed involved in mammalian corticogenesis.

Accordingly, we hypothesized that activation of AhR signaling is linked to cortical developmental in mice. In order to test this hypothesis, we selected a constitutively active (CA)-AhR-expressing transfection animal, because it was demonstrated that CA-AhR translocates into the nucleus and induces AhR target genes without ligands [37, 38] and that CA-AhR model animals manifest signs of dioxin toxicity, such as thymic involution, liver enlargement, and stomach cancer [39–41]. Thus, in this study, we used *in utero* electroporation [42, 43] to transfect CA-AhR in cortical projection neurons in the mouse brain and studied the significance of AhR signaling for cerebral cortical development.

## Materials and methods

### Reagents

Reagents were special grade and purchased from Wako Pure Chemicals (Osaka, Japan), unless otherwise stated.

### Animals

All animal experiments were performed using protocols approved by the Animal Care and Use Committee of the University of Tokyo (No. P12-41, PI = C. Tohyama) and that of Keio University [08065-(10), PI = K. Nakajima] in accordance with Institutional Guidelines on Animal Experimentation at the both universities as well as the Japanese Government Law concerning the Protection and Control of Animals and Japanese Government Notification of Feeding and Safekeeping of Animals. All efforts were made to reduce the total number of animals used and their suffering. Timed-pregnant C57BL/6N mice were purchased from Japan SLC (Shizuoka, Japan) for *in utero* electroporation experiments. These mice were housed in an animal facility maintained at 22°C−24°C and 40%−60% humidity under as 12/12 h light/dark cycle (lights on from 08:00 to 20:00). Laboratory rodent chow (Lab MR Stock; Nosan, Yokohama, Japan) and distilled water were provided *ad libitum*.

### Vector construction

**Fig 1:**
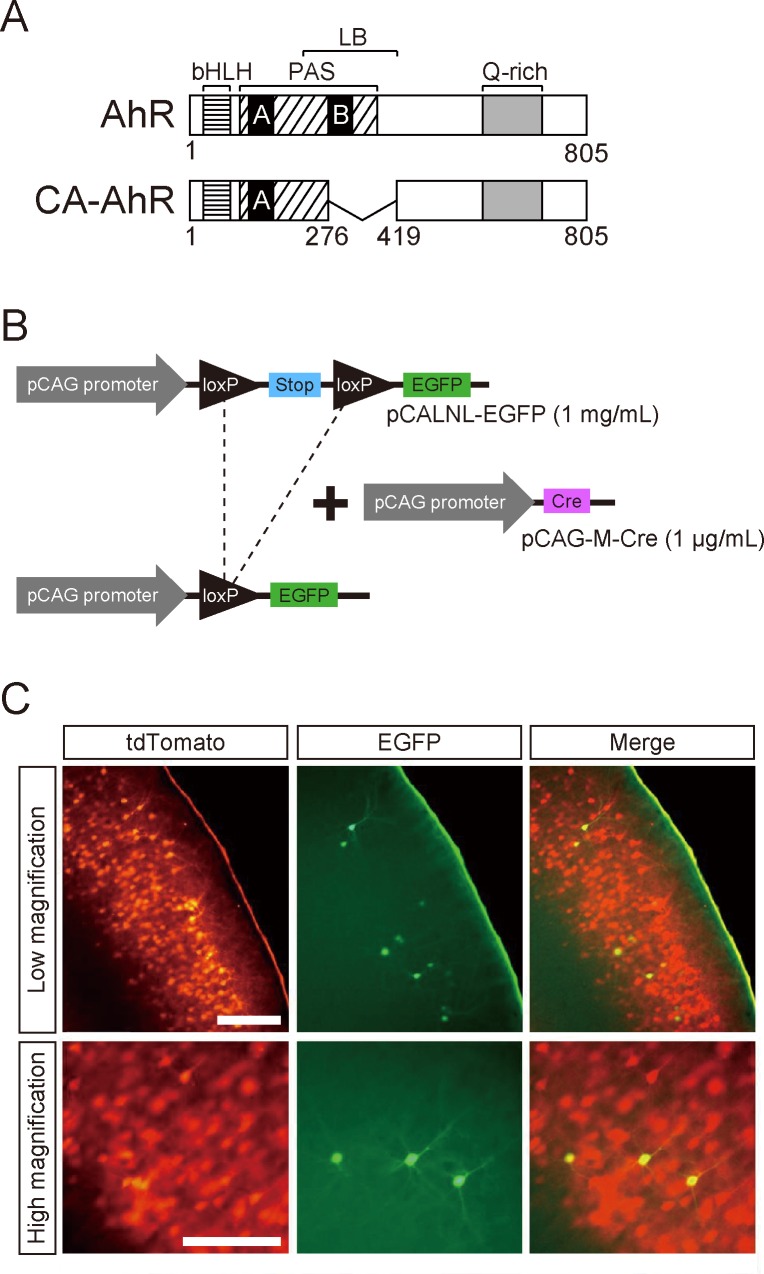
Sporadic expression of EGFP in pyramidal neurons of cerebral cortex using the Cre-LoxP recombination system. (A) Schematic diagrams of AhR and CA-AhR protein structures. Numbers represent amino acid positions. (B) Schematic diagram of Cre-mediated recombination with two kinds of plasmids (pCALNL-EGFP and pCAG-M-Cre) to obtain a plasmid that expresses EGFP. In the absence of pCAG-M-Cre, pCALNL-EGFP remains but does not express EGFP because of a SV40 polyadenylation (stop) sequence between the two loxP sites. To reduce the probability of recombination, a low ratio of pCAG-M-Cre to pCALNL-EGFP (1:1,000) was used, resulting in sparse EGFP-positive neurons among numerous tdTomato-positive neurons expressing AhR or CA-AhR for precise morphological analysis. (C) Representative photographs of fluorescent protein-expressing neurons in 14-day-old mouse somatosensory cortex transfected *in utero* (GD14.5). Scale bar = 200 and 100 μm at low and high magnification, respectively. bHLH, basic helix-loop-helix; LB, ligand-binding domain; PAS, Per-Arnt-Sim domain; Q-rich, glutamate-rich domain.

The AhR protein harbors several domains, including bHLH, PAS, ligand-binding, and Q-rich domains, comprising 805 amino acid residues, whereas CA-AhR lacks 142 amino acid residues spanning positions 277 to 418, a region including much of the ligand-binding domain (Fig 1A). Plasmid vectors pCAGGS1-AhR and pCAGGS1-CA-AhR were identical to those used in our previous study [37]. Briefly, a full-length AhR cDNA fragment was obtained from a mouse liver cDNA library by nested polymerase chain reaction (PCR) using two primer pairs: 5'-cctccgggacgcaggtg-3'/5'-agcatctcaggtacgggttt-3', and 5'-ctcgaggcgggcaccatgagcagcggcgcca-3'/5'-ctcgagtcaactctgcaccttgct-3'. A CA-AhR cDNA fragment was obtained by PCR amplification using pQCXIN-CA-AhR-EGFP as a template (a kind gift from Dr. Yoshiaki Fujii-Kuriyama, then at University of Tsukuba and Dr. Tomohiro Ito at the National Institute for Environmental Studies) and the specific primers 5'-ctcgaggcgggcaccatgagcagcggcgcca-3' and 5'-ctcgagtcaactctgcaccttgct-3'. The AhR and CA-AhR fragments were ligated into the pCRII-TOPO vector using the TOPO TA Cloning kit (Thermo Fisher Scientific, Carlsbad, CA, USA), amplified, and then excised by *Xho*I digestion for insertion into the *Xho*I site of pCAGGS1 to generate pCAGGS1-AhR and pCAGGS1-CA-AhR plasmids, respectively. These plasmids were subsequently used for *in utero* electroporation to induce AhR and CA-AhR expression in cortical pyramidal neurons. In a previous study, transfection of pCAGGS1-CA-AhR induced expression of the AhR targeted Cyp1a1 in neurons while pCAGGS1-AhR did not [37], suggesting that CA-AhR efficiently activates AhR downstream signaling in the absence of ligand.

### *In utero* electroporation

Pregnant mouse surgery and embryo manipulation *in utero* were performed as previously described [42–44]. Briefly, on gestational day (GD) 14.5, pregnant C57BL/6N mice were anesthetized with sodium pentobarbital (50 mg/kg b.w., *ip*) and laparotomized to expose the uterine horns. Embryos were divided into the following three groups depending on transfection condition: (1) a control group was transfected with a plasmid solution containing pCAGGS1-tdTomato, pCALNL-EGFP, and pCAG-M-Cre, (2) an AhR group with a plasmid solution containing pCAGGS1-tdTomato, pCALNL-EGFP, pCAG-M-Cre, and pCAGGS1-AhR, and (3) a CA-AhR group transfected with a plasmid solution containing pCAGGS1-tdTomato, pCALNL-EGFP, pCAG-M-Cre, and pCAGGS1-CA-AhR. The concentrations of pCAGGS1-tdTomato, pCAGGS1-AhR, pCAGGS1-CA-AhR, and pCALNL-EGFP were 1 mg/mL, and that of pCAG-M-Cre was 1 μg/mL.

For transfection of plasmid vectors, a total volume of 1−2 μL plasmid solution was injected into the lateral telencephalon ventricle using a glass micropipette and electroporated by placing a cathode adjacent to the neocortex. Using an electroporator (CUY21E; Nepa Gene, Chiba, Japan), electric pulses (30 V; 50 ms) were charged four times at every 950-ms interval. Injection of the plasmid vectors into the lateral telencephalon ventricle was monitored by including 0.1% Fast Green in the plasmid solution, and only embryos successfully injected were used in the present study. After electroporation, the uterine horns were placed back into the abdominal cavity to allow further embryonic growth and development.

### Preparation of brain tissues from electroporated mice

On postnatal day (PND) 14, mice transfected *in utero* were transcardially perfused with 4% paraformaldehyde (PFA; Nacalai Tesque, Kyoto, Japan) in 0.1 M phosphate-buffered saline (PBS, pH 7.4) under anesthesia with sodium pentobarbital. Brains were then collected, fixed in 4% PFA overnight, immersed in a series of solutions containing 5%, 15%, and 30% sucrose in 0.1 M PBS, frozen in Tissue-Tek O.C.T. compound (Sakura Finetek; Tokyo, Japan), and stored at −80°C. Frozen brains were sliced in the coronal plane through the somatosensory cortex using a cryostat (Model 3050S, Leica Microsystems, Tokyo, Japan). The somatosensory cortex of adult mouse brain is located between +0.26 mm and −1.94 mm from bregma [45]. Referring to this precedential information as well as our own morphology observations, the somatosensory cortex in 14-day-old mice was identified. Coronal sections of somatosensory cortex were cut at 150 μm thickness for dendritic morphology and at 50 μm thickness for neuronal positioning analyzes. Each tissue slide was covered with Vectashield, counterstained with DAPI (Vector Laboratories, Burlingame, CA, USA), and sealed under a cover glass.

### Dendritic morphology analysis

To reveal complete dendritic morphology under fluorescence microscopy (Model DM6000 B, Leica Microsystems), all transfection solutions contained high concentrations of pCALNL-EGFP and pCAGGS1-tdTomato (1 mg/mL each) and a low concentration of pCAG-M-Cre (1 μg/mL) for sporadic EGFP expression throughout the entire dendritic structure within a larger field of tdTomato-stained neurons (Fig 1B). Indeed, numerous tdTomato-expressing neurons and a small number of Cre-dependent EGFP-expressing neurons were observed in cortical layer II/III of the electroporated mouse brain (Fig 1C). Dendritic morphology of EGFP-expressing neurons was analyzed using the Neurolucida tracing system (MicroBrightField, Colchester, VT, USA). A single neuron was traced under a microscope equipped with a specific objective lens (Leica DM6000 B, HCX PL APO, 40×, NA = 0.75; Leica Microsystems) by an observer blinded to the transfection group. Three-dimensional dendritic morphology was quantified using NeuroExplorer software (MicroBrightField). Reconstructed dendrites were analyzed for arbor complexity, length, and number of branching points. The complexity of dendrites in control, AhR, and CA-AhR group neurons was determined by Sholl analysis; counting the number of intersections with concentric rings (Sholl rings) defined every 10 μm between 20 and 200 μm from the cell body. For each mouse, 15 to 20 neurons were subject to Sholl ring analysis for a total of 54 control, 47 AhR, and 66 CA-AhR group neurons from 3–4 mice/group.

### Neuronal positioning analysis

Neuronal positioning in the cerebral cortex was evaluated using bin analysis as described previously [46]. Briefly, the cortex image was equally divided into 10 bins, with the bins closest to the ventricle and pia mater defined as bin 1 and bin 10, respectively. In each bin, the number of tdTomato-expressing neurons was estimated as a percentage of the total number in all 10 bins using ImageJ software (National Institute of Health, Bethesda, MD, USA). The percentage of neuronal distribution in each bin was compared between the control and CA-AhR groups (n = 3 and 5 mice group, respectively).

### Gene expression analysis

Brain slices collected from successfully transfected mice on PND 14 were placed on steel-framed PPS membrane slides (Leica Cat. No. 11505268). The tdTomato-positive areas of the somatosensory cortex were chosen as regions of interests (ROIs), and excised from slices using a laser microdissection (LMD) microscope (Model LMD7000, Leica Microsystems). Excised samples of somatosensory cortex were 50 μm thick and ranged from 922,821 to 2,683,471 μm^2^ in surface area. Samples were prepared for RNA quantification as described previously [47]. In brief, total RNA was extracted using a CellAmp Direct RNA Prep Kit lysis buffer (Takara Bio, Kusatsu, Japan) containing proteinase K (0.3 U, Takara Bio). This solution was incubated at 50°C for 30 min, and subjected to sonication for 1 min. Proteinase K was inactivated by incubation of RNA extract solution at 75°C for 5 min. Extracts were then treated with DNase (0.05 U, Takara Bio) at 37°C for 5 min, followed by incubation at 75°C for 5 min for DNase inactivation. RNAs were reverse-transcribed using PrimeScript (Takara Bio) with both oligo-dT and random N_6_ primers according to the manufacturer’s instructions. RNA extracts from three control and five CA-AhR group mice were subjected to quantitative real-time RNA expression analysis. The quantitative detection of cDNAs was performed by SYBR Green I-based real-time PCR using a Light Cycler instrument (Roche Molecular Biochemicals, Manheim, Germany) and Thunderbird SYBR qPCR mix (Toyobo, Osaka, Japan). Negative controls for qPCR were analyzed concomitantly to confirm that the samples were not cross-contaminated. The expression levels of RNAs (mRNAs and rRNA) are presented as crossing number normalized to *18S rRNA* expression levels, as it has been demonstrated that the amount of *18S rRNA* linearly correlates with the size of the ROI. Primer pairs for real-time qPCR are shown in Table 1.

**Table 1:** Primer sequences for LMD-RT-qPCR.

Gene symbol	Forward primer	Reverse primer
*Cyp1a1*	5ꞌ-caccgtattctgccttggat-3ꞌ	5ꞌ-cagcatgtgaccaatgaagg-3ꞌ
*Cyp1b1*	5ꞌ-ggacaaggacggcttcatta-3ꞌ	5ꞌ-gcgaggatggagatgaagag-3ꞌ
*Ahrr*	5ꞌ-cagggcagacattgtggtta-3ꞌ	5ꞌ-ctccattgctctttcctgct-3ꞌ
*Gapdh*	5ꞌ-acccagaagactgtggatgg-3ꞌ	5ꞌ-cacattgggggtaggaacac-3ꞌ
*18S rRNA*	5ꞌ-ggaccagagcgaaagcatttg-3ꞌ	5ꞌ-ttgccagtcggcatcgtttat-3ꞌ

### Statistical analysis

Dendritic length and branching point numbers were compared between transfection groups using one-way analysis of variance (ANOVA) followed by Tukey–Kramer *post hoc* test for pair-wise comparisons. Sholl and bin analysis data were statistically examined using two-way ANOVA followed by Tukey–Kramer *post hoc* test and Student’s *t*-test, respectively. Gene expressions were statistically analyzed using Student’s *t-*test. In all tests, *P*-values <0.05 were considered statistically significant.

## Results

### CA-AhR overexpression reduces the morphological complexity of cortical pyramidal neuron dendrites

The apical and basal dendrites of EGFP-expressing pyramidal neurons in cortical layer II/III were traced in 14-day-old mice that were electroplated *in utero* with AhR or CA-AhR plasmid. Dendritic complexity was compared among control, AhR, and CA-AhR groups using five metrics: number of intersections with concentric rings (Sholl rings) surrounding the soma, entire dendritic length, number of branching points, and the length and number of the first- to fifth-order branches.

**Fig 2:**
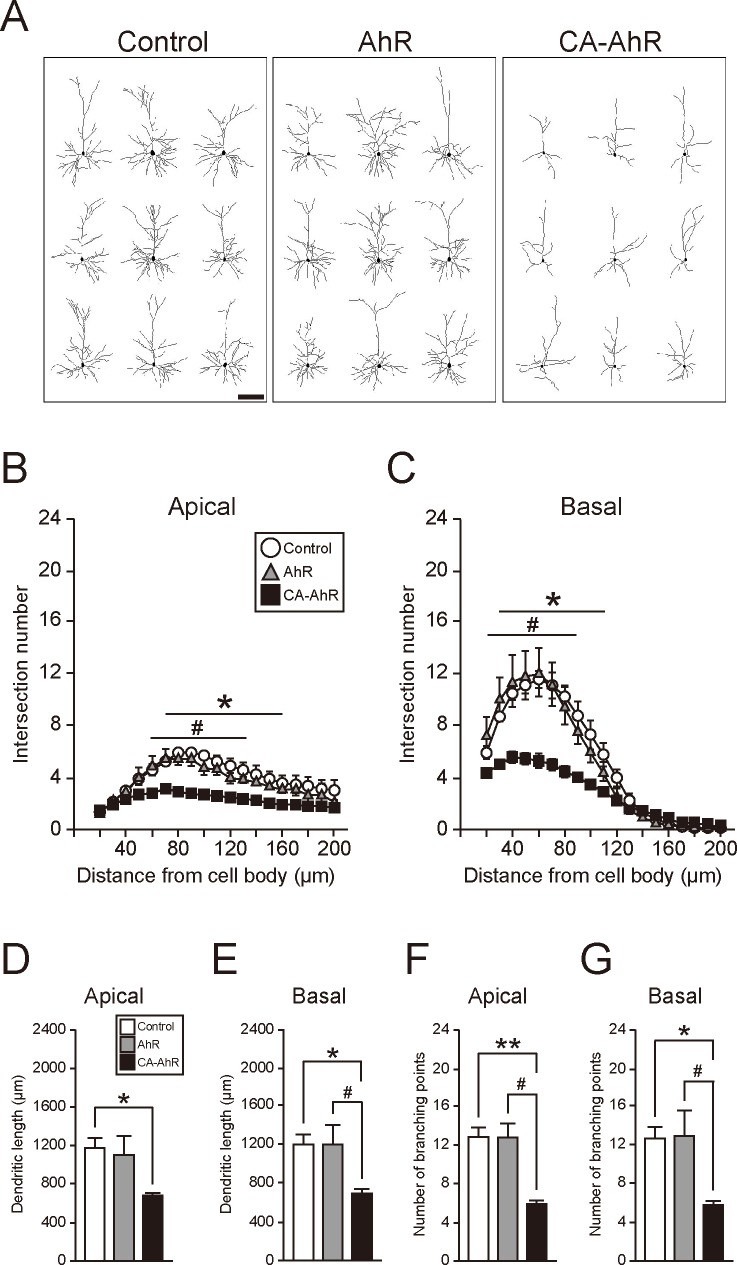
Overexpression of constitutively active-AhR (CA-AhR) reduces the morphological complexity of somatosensory cortex layer II/III pyramidal cell dendrites. (A) Representative drawings of EGFP-expressing pyramidal neurons in cortical layer II/III of 14-day-old mice. Neurons with the characteristic morphology of CA-AhR, AhR, and control groups were randomly chosen for display (n = 3–4/group). A distinct reduction in dendritic arborization in the CA-AhR group was observed compared to control mice and mice transfected with AhR. (B−G) Quantification of dendritic complexity (B, C), length (D, E), and number of branching points (F, G) of apical (B, D, F) and basal (C, E, G) dendrites. Values are mean ± S.E.M. from 3 control, 3 AhR, and 4 CA-AhR group mice. Asterisks denote significant differences between the control and CA-AhR groups, whereas sharps indicate significant differences between the AhR and CA-AhR groups. Single (*, #) and double (**) symbols denote significance by Tukey–Kramer *post hoc* test at *p* < 0.05 and 0.01, respectively. Scale bar = 100 μm.

Dendritic arborization was markedly reduced in the CA-AhR group compared to control and AhR groups (Fig 2A). In both apical and basal dendrites, two-way repeated measures ANOVA indicated significant main effects of vector-transfection group (*F*(2, 133) = 115, *p* = 8.80 × 10^−30^; *F*(2, 133) = 83.7, *p* = 2.93 × 10^−24^, respectively) and distance from the cell body (*F*(18, 133) = 15.9, *p* = 6.38 × 10^−25^; *F*(18, 133) = 84.2, *p* = 1.69 × 10^−63^, respectively), and a significant interaction between these factors (*F*(36, 133) = 1.71, *p* = 0.0158; *F*(36, 133) = 6.20, *p* = 3.33 × 10^−15^, respectively). The number of apical dendrite intersections of the CA-AhR group was significantly lower at each Sholl ring between 70 and 160 μm from the cell body than that of the control group (*p* < 0.05) and between 60 and 130 μm from the cell body than that of the AhR group (*p* < 0.05; Fig 2B). Similarly, the number of basal dendrite intersections of the CA-AhR group was significantly lower at each Sholl ring between 30 and 110 μm from the cell body than that of the control group (*p* < 0.05) and between 20 and 90 μm from the cell body than that of the AhR group (*p* < 0.05; Fig 2C).

One-way ANOVA also indicated significant differences in entire apical and basal dendritic lengths among groups (*F*(2, 7) = 5.48, *p* = 0.0369; *F*(2, 7) = 7.01, *p* = 0.0213, respectively). Apical dendritic length was significantly shorter in the CA-AhR group than in the control group (*p* < 0.05; Fig 2D). Moreover, basal dendrite length was significantly shorter in the CA-AhR group than in both control and AhR groups (*p* < 0.05 and 0.05, respectively; Fig 2E). One-way ANOVA indicated significant differences in the number of branching points along both apical and basal dendrites among groups (*F*(2, 7) = 11.3, *p* = 0.00646; *F*(2, 7) = 7.52, *p* = 0.0181, respectively). The number of apical dendrite branching points was significantly lower in the CA-AhR group than in the control and AhR groups (*p* < 0.01 and 0.05, respectively; Fig 2F). Similarly, the number of basal dendrite branching points in the CA-AhR group was significantly lower than that in the control and AhR groups (*p* < 0.05 and 0.05, respectively; Fig 2G).

To analyze the length and number of branches of apical and basal dendrites, we used the first- to fifth-order branches, because the number of branching points in the CA-AhR-transfected neurons was found to be significantly reduced, which caused a drastic decrease in higher-order branches. In apical dendrites, one-way ANOVA indicated significant differences between groups in the lengths of the first- (*F*(2, 7) = 6.23, *p* = 0.0279), second- (*F*(2, 7) = 9.28, *p* = 0.0107), and third-order (*F*(2, 7) = 20.0, *p* = 0.00127) branches, but not the fourth- (*F*(2, 7) = 0.934, *p* = 0.437) and fifth-order (*F*(2, 7) = 2.81, *p* = 0.127) branches (S1 Fig). The first-order branches of the CA-AhR group were significantly longer than that of the control group (*p* < 0.05), while the second- and third-order branches were significantly longer than those of both the control (*p* < 0.05 and 0.01, respectively) and AhR groups (*p* < 0.05 and 0.01, respectively). Conversely, in basal dendrites, one-way ANOVA also indicated significant differences in the second- (*F*(2, 7) = 55.0, *p* = 5.24 × 10^−5^) and fifth-order (*F*(2, 7) = 10.4, *p* = 0.00793) branches, but not in the first- (*F*(2, 7) = 4.11, *p* = 0.660), third- (*F*(2, 7) = 0.298, *p* = 0.752), and fourth-order (*F*(2, 7) = 2.05, *p* = 0.198) branches (S1 Fig). The second-order branches of the CA-AhR group were significantly longer than those of the control (*p* < 0.01) and AhR groups (*p* < 0.01), and the fifth-order branches were significantly shorter than those of the control (*p* < 0.05) and AhR groups (*p* < 0.01). Next, we analyzed and compared the first- to fifth-order branch numbers among the control, AhR, and CA-AhR groups. One-way ANOVA indicated significant differences among groups in the third- (*F*(2, 7) = 7.65, *p* = 0.0173), fourth- (*F*(2, 7) = 7.87, *p* = 0.0162), and fifth-order (*F*(2, 7) = 13.4, *p* = 0.00406) apical branches, but not the first- and second-order branches. The first-order apical branch is always single, and the second-order apical branch has usually two or occasionally three branches in the all three groups (S1 Fig). The numbers of the third-, fourth-, and fifth-order apical branches were significantly lower in the CA-AhR group than in the control (*p* < 0.05, 0.05, and 0.01, respectively) and AhR (*p* < 0.05, 0.05, and 0.05, respectively) groups. One-way ANOVA indicated significant differences among groups in the numbers of the first- (*F*(2, 7) = 8.31, *p* = 0.0142), second- (*F*(2, 7) = 6.66, *p* = 0.0240), third- (*F*(2, 7) = 14.4, *p* = 0.00328), and fourth-order (*F*(2, 7) = 6.09, *p* = 0.0294) basal branches, but not the fifth-order (*F*(2, 7) = 3.16, *p* = 0.105) basal branches (S1 Fig). The numbers of the third- and fourth-order basal branches were significantly lower in the CA-AhR group compared to the control group (*p* < 0.01 and 0.05, respectively). In addition, the numbers of the first-, second-, and third-order basal branches was significantly lower in the CA-AhR group compared to the AhR group (*p* < 0.05, 0.05, and 0.05, respectively). These results suggest that CA-AhR transfection impairs the process of dendritic branching formation and then elicits a decrease in the entire length and number of dendrites (Fig 2D–2G). Although the entire dendrite length was significantly decreased in the CA-AhR group, branch lengths of apical (the first, second, and third orders) and basal (the second order) dendrites were increased (S1 Fig). These increases are considered to be caused by the loss of dendritic branching points at specified-order branches.

**Fig 3:**
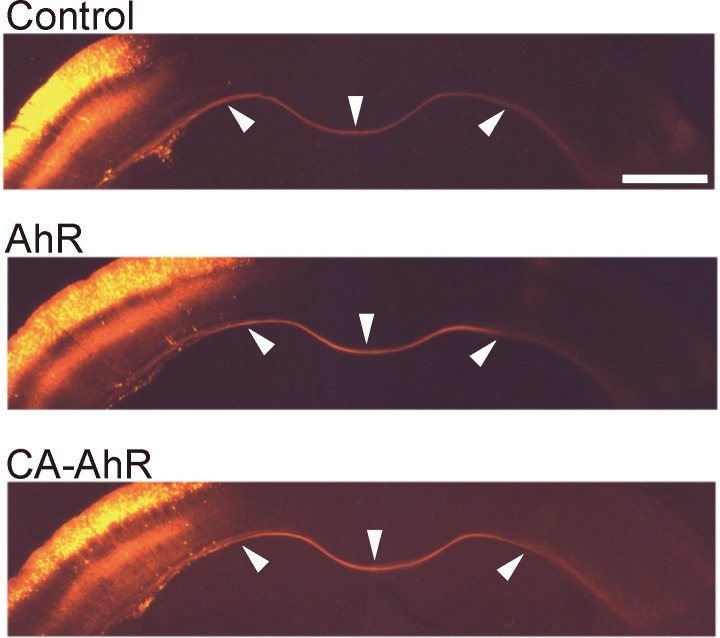
Axonal projection is unaffected by CA-AhR. Representative photographs of commissural fibers of tdTomato-expressing pyramidal neurons in cortical layer II/III of 14-day-old mice. No difference in axonal projection pattern was observed among the control, AhR, and CA-AhR groups. Arrowheads (left, middle, and right) indicate commissural fibers in the transfected hemisphere, medial line, and contralateral hemisphere, respectively. Scale bar = 1.0 mm.

In contrast, no conspicuous differences in the projection pattern of commissural fibers was observed in the CA-AhR group compared with the control and AhR groups, suggesting that CA-AhR preferentially impairs dendritic growth rather than axonal elongation (Fig 3).

### CA-AhR overexpression affects neuronal positioning

**Fig 4:**
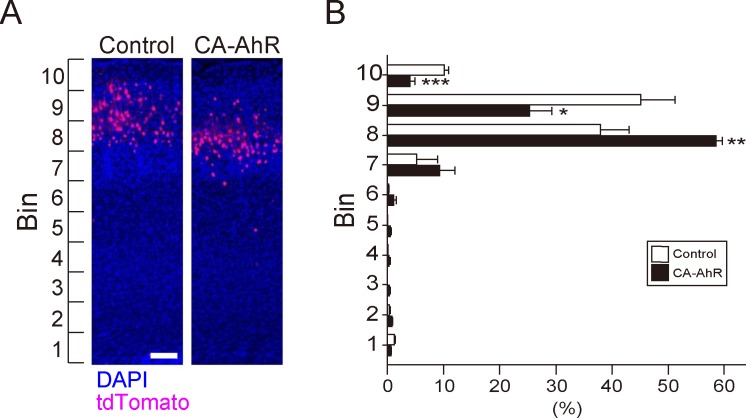
Altered distribution of neurons overexpressing CA-AhR. (A) Representative photographs of tdTomato-expressing (red) neurons in the control and CA-AhR groups. Blue background is DAPI staining. Scale bar = 100 μm. (B) The distribution of tdTomato-expressing cortical neurons in each bin. Values are mean ± S.E.M from 3 control and 5 CA-AhR group mice. Asterisks denote significance differences between the control and CA-AhR groups. Single (*), double (**), and triple symbols (***) denote significant difference by Student’s *t*-test at *p* < 0.05, 0.01, and 0.001, respectively.

To further investigate effects of CA-AhR on development of the cerebral cortex, we examined the positions of tdTomato-expressing neurons in the cortical gray matter by bin counting analysis. Transfection of CA-AhR, but not AhR, caused distinct abnormalities in dendritic morphology, so we examined neuronal distribution from the cortical surface (bin 10) to the ventricular wall (bin 1) only for the control and CA-AhR groups (Fig 4A). Two-way repeated measures ANOVA indicated a significant difference in neuronal distribution in each bin (*F*(9, 60) = 159, *p* = 1.95 × 10^−38^) and a significant interaction of vector-transfection with neuronal distribution in bins 1−10 (*F*(9, 60) = 11.3, *p* = 3.87 × 10^−10^). Student’s *t*-test indicated that the percentage of tdTomato-expressing neurons in the CA-AhR group was significantly higher in bin 8 (*F*(1, 6) = 29.0, *p* < 0.01) and lower bin 9 (*F*(1, 6) = 8.23, *p* < 0.05) and 10 (*F*(1, 6) = 36.6, *p* < 0.001) compared to the control group (Fig 4B). These results suggest that CA-AhR causes a defect in neuronal positioning during cortical development.

### CA-AhR induces expression of AhR target genes in transfected cortex

**Fig 5:**
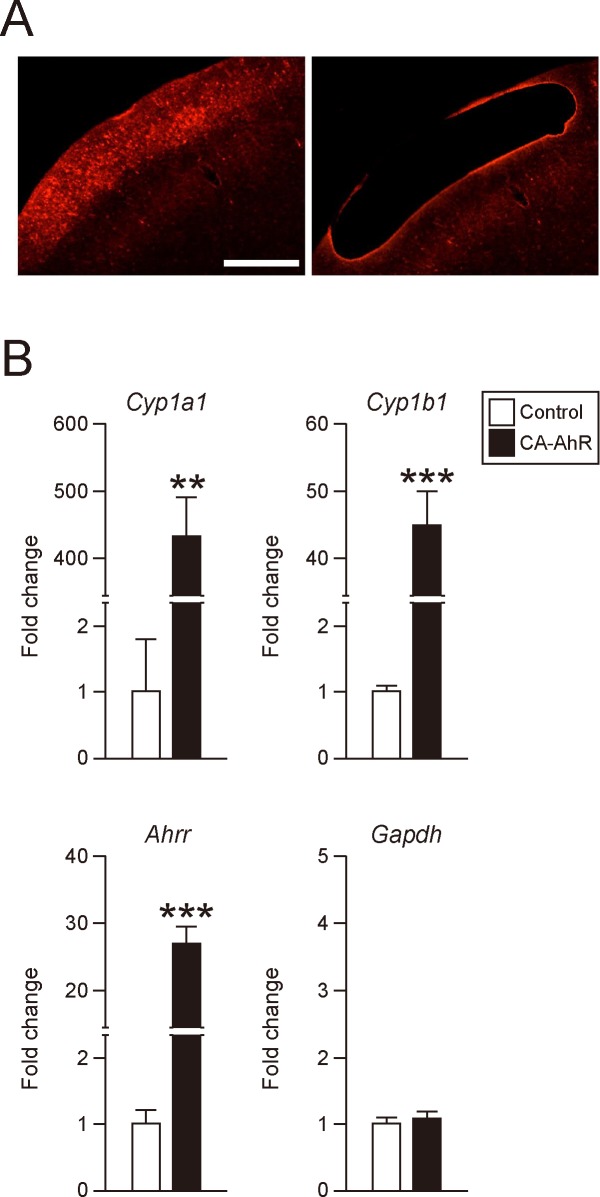
Expression analysis of AhR target genes in tdTomato-transfected cerebral cortex. (A) Representative photographs of cerebral cortex section before (left) and after (right) microdissection of the tdTomato-positive area. Scale bar = 500 μm. (B) Quantitative real-time RT-PCR analysis of *Cyp1a1*, *Cyp1b1*, *Ahr repressor* (*Ahrr*), and *Gapdh* mRNA expression levels in the tdTomato-positive area. Quantitative fold changes were normalized to *18S rRNA* expression levels. Values are mean ± S.E.M. from 3 control and 5 CA-AhR group mice. Asterisks (** and ***) denote significant differences by Student’s *t*-test at *p* < 0.01 and 0.001, respectively.

To confirm that CA-AhR activates AhR signaling pathways in this model, expression levels of the AhR target genes *Cyp1a1*, *Cyp1b1*, and *Ahrr* were measured in tdTomato-positive cortical regions isolated from somatosensory cortex of the CA-AhR and control groups by LMD microscopy (Fig 5A). The expression levels of *Cyp1a1* (*F*(1, 6) = 34.5), *Cyp1b1* (*F*(1, 6) = 41.1), and *Ahrr* (*F*(1, 6) = 61.9) mRNA levels were significantly increased in the CA-AhR group compared with the control group (Student’s *t*-test, *p* < 0.01, 0.001, and 0.001, respectively; Fig 5B). Alternatively, there was no significant difference in expression of *Gapdh*, a housekeeping gene used as a negative control, between the control and CA-AhR groups (*F*(1, 6) = 0.553, *p* = 0.485). These results suggest that CA-AhR can function as a transcription factor to induce AhR signaling in transfected neurons.

## Discussion

In the present study, we demonstrate that constitutively activated AhR signaling impairs dendritic growth and positioning of pyramidal neurons in mouse cortical layer II/III (Fig 2, S1 Fig). While bHLH family members (including bHLH only, bHLH-Zip, and bHLH-PAS members) have been implicated in the proliferation, migration, dendritic growth, and neuronal differentiation during mammalian brain development [7–15], physiological functions of AhR in developing mammalian cortex have been obscure. Previous studies have linked AhR signaling to neuronal differentiation, neuronal migration, axonal branching, and dendritic arborization in *C*. *elegans* and *Drosophila* [21–23] as well as to the regulation of dendritic morphology in subneocortical hippocampal pyramidal and olfactory granule neurons [37, 48] and to cerebellar neurogenesis [24]. Expression of AhR transcripts has been reported in rodent brain regions, including the cerebral cortex and hippocampus [16, 17]. Abnormal dendritic morphology of hippocampal CA1 neurons was shown in both TCDD-exposed and CA-AhR-transfected mice [37], suggesting that AhR is expressed in hippocampal pyramidal neurons and that its signaling is associated with dendritic growth. The present study also shows abnormal dendritic morphology in CA-AhR-transfected pyramidal neurons in the cerebral cortex (Fig 2; S1 Fig); therefore, it is plausible that AhR may be expressed in not only the hippocampus but also the cerebral cortex. In the present study, we further revealed that AhR signaling regulates dendritic arborization and neuronal positioning in mammalian neocortex and suggest that these effects may explain the behavioral impairments caused by dioxin. CA-AhR transfection in the present study may mimic AhR over-activation by dioxin. Animals born to dioxin-exposed dams exhibited abnormalities in higher brain function [29–35], possibly due to impaired dendritic morphology [37], cortical layer malformation [49], and/or impaired cerebellar neurogenesis [50]. It is plausible that impaired dendritic growth and migration of cortical pyramidal neurons also contributes to these cognitive and behavioral abnormalities in dioxin-exposed animals. This speculation is parallel to the previous findings in which CA-AhR mouse models develop stomach tumor [39], liver enlargement [40], and thymic involution [41]. In the present study, expression of AhR target genes was drastically elevated in CA-AhR-transfected cortex (Fig 5), suggesting that CA-AhR-transfected model mimics the situation of high-dose exposure to AhR agonist. In accordance with this notion, significant increase in Cyp1a1 transcripts has been shown in several brain regions, including the cerebral cortex in TCDD-exposed rats [51]. On the other hand, in our previous study, no significant alteration in Cyp1a1 expression was observed in the cerebral cortex of mouse offspring born to dams administered TCDD [36]. Therefore, CA-AhR is considered to be a useful model to elucidate molecular mechanisms of AhR signaling in cellular physiology; however, a limitation of CA-AhR transfection study is that it is not possible to regulate the expression level of AhR in target neurons, and that the quantity of activated AhR in the CA-AhR mouse models may be far higher than the level in the AhR transfection group. In addition, there was no obvious alteration in axonal elongation in the CA-AhR group (Fig 3), suggesting that dendritic growth is more susceptible to AhR signaling activation rather than axonal elongation.

Dendritic growth of pyramidal neurons is regulated by a complex signaling pathway in a cortical layer-specific manner. Neurotrophins such as BDNF and NT-3 enhanced the growth of both apical and basal dendrites in ferret cortical layer IV to VI [52], while in rodents, BDNF and NT-3 promote branching of basal dendrites, but not apical dendrites, in cortical layer II/III [53]. Insulin-like growth factor 1 (IGF-1) increases branching of both apical and basal dendrites in rat cortical layer II/III [53]. Additionally, Sema3A has been reported to elongate apical dendrites, but not basal dendrite, of pyramidal neurons in layer V of the rodent cortex [54]. Among these molecules, it is most likely that CA-AhR disrupts IGF-1-mediated signaling because morphological changes by CA-AhR were observed in both apical and basal dendrites in cortical layer II/III neurons (Fig 2, S1 Fig). Indeed, exposure to dioxin has been reported to inhibit the IGF-1 signaling pathway [55].

In the present study, CA-AhR transfection disrupted neuronal positioning in the cerebral cortex (Fig 4). Because AhR-transfected hippocampal neurons were found not to alter neuronal positioning [56], it is feasible to speculate that neuronal positioning in the cerebral cortex could not be altered in the AhR group. In cortical projection neurons, “late-born” neurons migrate radially toward the brain surface and pass “early-born” neurons, which forces the early-born neurons to be aligned in the deeper layers, the phenomenon of which is expressed as an “inside-out” manner [57]. Percentage of transfected neurons in the CA-AhR group was increased in deeper layers (Fig 4), suggesting that cell division was initiated early by AhR signaling. In addition, cell cycle exit was promoted in cortical layer V-VI neurons of mice perinatally exposed to TCDD [49]. Hence, it is plausible that AhR signaling may affect neuronal migration through perturbed neurogenesis in the developing cortex.

Since CA-AhR disrupted fine structure of both apical and basal dendrites (Fig 2, S1 Fig), it is plausible that AhR signaling is involved in some processes fundamental to the growth of all dendrites, such as cytoskeletal dynamics. Indeed, AhR loss- and gain-of-function experiments have shown that AhR regulates polymerization and depolymerization of actin fibers. Further, an *in vitro* study showed increased actin fibers in AhR-null fibroblasts [58]. Exposure to dioxin also promoted actin remodeling in MCF7 and HepG2 cells [59, 60], and CA-AhR expression enhanced actin remodeling in MCF7 cells [59]. These results suggest a substantial role for AhR signaling in actin dynamics, while over-activation may disrupt normal cytoskeletal function, resulting in impaired morphological development and migration. During migration, cell shape and motility are controlled by secreted and intracellular molecules that regulate actin dynamics. For example, Reelin is a secreted protein that regulates neuronal migration during cortical development [61], presumably via cofilin-mediated actin stabilization [62]. In addition, Disc1 acts as a scaffold protein and regulates tangential migration of inhibitory neurons in the developing cerebral cortex via stabilization of actin filaments [63]. Taken together, over-activation of AhR signaling by CA-AhR may perturb cytoskeleton regulation, which subsequently disrupts dendritic growth and/or migration of cortical pyramidal neurons during development.

## Supporting information

S1 Fig Differences in specific order branch numbers and lengths in neurons overexpressing CA-AhR. Lengths (A, B) and number (C, D) of the first- to fifth-order branches of apical (A, C) and basal (B, D) dendrites. Values are mean ± S.E.M. from 3 control, 3 AhR, and 4 CA-AhR group mice. (E) Scheme of impaired dendritic growth of CA-AhR-transfected neurons. Symbols (asterisks and sharps) denote significant differences between the control and CA-AhR groups, and between the AhR and CA-AhR groups, respectively. Single (*, #) and double (**, ##) symbols denote significant difference by Tukey–Kramer *post hoc* test at *p* < 0.05 and 0.01, respectively.
(EPS)
